# Kidneys in Children with Tuberous Sclerosis Complex—An Up-to-Date Review

**DOI:** 10.3390/jcm14217805

**Published:** 2025-11-03

**Authors:** Anna Maria Wabik, Jakub Pytlos, Aneta Michalczewska, Piotr Skrzypczyk

**Affiliations:** 1 Department of Pediatrics and Nephrology, Medical University of Warsaw, 02-091 Warsaw, Poland; annamariawabik@gmail.com (A.M.W.); aneta.michalczewska@wp.pl (A.M.); piotr.skrzypczyk@wum.edu.pl (P.S.); 2 Department of Pediatric Radiology, Medical University of Warsaw, 02-091 Warsaw, Poland; 3 2nd Department of Clinical Radiology, Doctoral School, Medical University of Warsaw, 02-091 Warsaw, Poland

**Keywords:** tuberous sclerosis complex, renovascular hypertension, diagnostic imaging, angiomiolipoma, chronic kidney disease, magnetic resonance imaging, ultrasound

## Abstract

**Background**: Tuberous sclerosis complex (TSC) is a rare genetic disorder characterized by the growth of benign tumors in various organ systems, with particularly significant effects on the kidneys. Renal manifestations of TSC include angiomyolipomas (AMLs), renal cysts, and a higher risk of renal cell carcinoma (RCC). Nephrological monitoring is crucial for the early detection of kidney changes, the management of hypertension, and the assessment of the risk of developing chronic kidney disease. Ultrasound is typically the initial imaging choice for diagnosis and monitoring, with magnetic resonance imaging (MRI) being a preferred imaging modality for long-term surveillance. Patients with TSC have an increased risk of arterial hypertension, renal artery stenosis, and urolithiasis. In some patients, the co-occurrence of TSC and autosomal dominant polycystic kidney disease (ADPKD) is caused by the TSC2/PKD1 contiguous gene syndrome (CGS). The primary medical treatment for TSC is a mammalian target of rapamycin kinase inhibitors (mTOR), as they effectively shrink tumors, often reducing or eliminating the need for surgical intervention. **Methods**: This article aims to review the most recent literature on the diagnosis and management of renal lesions in tuberous sclerosis complex (TSC), with a particular focus on the role of various imaging techniques. **Conclusions**: Given the multifactorial nature of this disease, this review emphasizes the importance of a multidisciplinary approach, including various imaging methods, to improve the care and treatment outcomes of children with tuberous sclerosis complex.

## 1. Introduction

Tuberous sclerosis complex (TSC) is a rare, autosomal dominant genetic, multisystem disease caused by a mutation in tumor suppressor genes: TSC1 (chromosome 9q34) and TSC2 (chromosome 16p13), with an incidence rate from 1:6760 to 1:13,520 live births [1]. The prevalence of TSC varies—among the Caucasian population, it ranges from 1 in 7872 (Quebec, Canada) to 1 in 24,956 (Northern Ireland). In the Asian population, the most frequent studies were conducted in Taiwan, 1 in 63,291 in 2010 [2]. It is estimated that 2 million people worldwide suffer from tuberous sclerosis [3].

The TSC1 and TSC2 genes encode the proteins hamartin and tuberin, respectively, which function as inhibitors of the mammalian target of rapamycin (mTOR) signaling pathway. Uncontrolled activation of the mTOR pathway results in the development of hamartomas, benign neoplasms, and, rarely, malignant neoplasms in multiple organs, including the brain, lungs, heart, kidneys, and skin [4]. Mutations in the TSC2 gene, which occur in 80–90% of cases, are more prevalent and associated with a more severe clinical phenotype [5]. In addition, the proximity of the TSC2 gene to the PKD1 gene, mutations of which cause polycystic kidney disease (PKD), results in a contiguous gene syndrome (CGS). Approximately 2% of individuals with TSC also have PKD [4,5]

## 2. Manifestations and Diagnosis of Tuberous Sclerosis Complex

The updated International Tuberous Sclerosis Complex Diagnostic Criteria and Surveillance and Management Recommendations by H. Northrup et al. reinforce the importance of independent genetic and clinical diagnostic criteria (Table 1) [6]. Identification of a pathogenic variant in TSC1 or TSC2 is sufficient for diagnosing or predicting TSC regardless of clinical findings; when interpreted according to the standards and guidelines of the American College of Medical Genetics (ACMG) for interpreting sequence variants, which are now widely adopted as the international standard [7]. A failure to identify a pathogenic variant in TSC1 or TSC2 does not exclude a diagnosis of TSC. The updated diagnostic clinical criteria include eleven major and seven minor features. A definitive diagnosis of TSC is made when an individual presents with two or more major features or one major feature and at least two minor features. A possible diagnosis of TSC is considered when an individual exhibits one major feature or two or more minor features.

Clinical features of TSC are diverse, ranging from barely noticeable, mildly symptomatic to severe forms and involve multiple systems, involving abnormalities of the skin (hypomelanotic macules, confetti skin lesions, facial angiofibromas, shagreen patches, fibrous cephalic plaques, ungual fibromas); brain (subependymal nodules, cortical tubers, and subependymal giant cell astrocytomas [SEGAs], epilepsy, TSC-associated neuropsychiatric disorder [TAND]); eyes (retinal hamartomas, chorioretinal hypopigmentation), kidneys (benign renal angiomyolipomas, epithelial cysts, oncocytoma, renal cell carcinoma); heart (rhabdomyomas, arrhythmias); lungs (lymphangioleiomyomatosis [LAM], multifocal micronodular pneumonocyte hyperplasia), liver (hepatomegaly, angiomyolipomas, lipomas, hamartomas, and fibromas), pancreas (pancreatic neuroendocrine tumors [PanNETs]), and skeletal abnormalities (cyst-like lesions, hyperostosis of the inner table of the calvaria, osteosclerotic changes, periosteal new bone formation, cystic changes in the phalanges, and scoliosis [8,9,10].

## 3. Renal Manifestations of Tuberous Sclerosis Complex in Children

There are several types of renal tumours associated with TSC syndrome, including angiomiolipomas (AMLs), including renal cysts, oncocytomas and renal cell carcinoma (RCC) [11].

**Figure 1:**
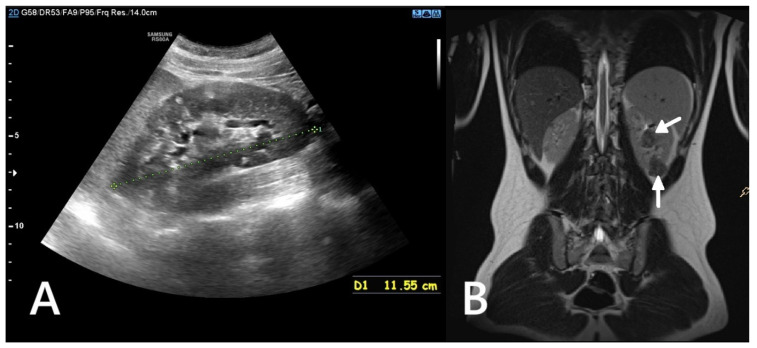
Ultrasound (**A**) and MRI (**B**) images of a 16-year-old boy. The ultrasound reveals numerous hyperechoic lesions, consistent with AMLs, along with several cysts (**A**). MRI shows large focal lesions (arrows), most likely representing atypical AMLs (**B**).

Renal involvement in children with TSC is estimated to occur in approximately 80% of cases. The most common kidney manifestations are angiomyolipomas (70–80%) and cysts (around 50%), presented in Figure 1. Other tumors, including RCC or renal oncocytoma, are less common (<2%) [12,13,14].

### 3.1. Angiomyolipoma

Angiomyolipoma (AML) is a mesenchymal neoplasm caused by insufficient or defective activation of the TSC1 or TSC2 genes, which leads to uncontrolled activation of the mammalian target of rapamycin (mTOR) pathway [15]. Hemangiomas occur in approximately 20% of children with TSC aged <2 years and in 67% of children with TSC aged up to 10 years [16]. These lesions are observed less frequently in children under 2 years of age, likely because of their slow growth and small size, often not seen in imaging studies. The size of the AMLs has been shown to accelerate with age up to 1.03 mm/year (age 5–9 years), 2.29 mm/year (age 9–14 years) and 2.82 mm/year (age 15–19 years) [17]. Hormone receptors (for estrogen and progesterone) are present on the surface of AMLs, so peri-pubertal phases, pregnancy or hormone treatment can potentially increase the growth of AML due to the amplifying effect of these hormones [18].

AMLs are classified as classical (typical) AML, fat-poor (atypical) AML, and epithelioid AML. AML is likely to originate from perivascular epithelial cells (PECs) and is also referred to as PECOMA or at least considered part of the PECOMA family.

AMLs are localized in the renal parenchyma and not in the capsule or perinephric tissue. Histologically, a typical angiomyolipoma consists of three components: vessels (angio); spindle cells (myo) and adipose tissue (lipo). The vessels are typically eccentric and thick-walled. Spindle-shaped tumor cells may grow around them, exhibiting both smooth muscle and melanocytic features. These include mature smooth muscle cells and immature spindle-shaped epithelioid cells. Adipocytes, interspersed with spindle cells, are mature and lack cytological atypia. Immunohistochemically, the spindle cell component of AML often shows positivity for melanocytic markers such as HMB-45 and Melan-A, as well as smooth muscle markers like smooth muscle actin. In contrast, keratins and other epithelial markers are consistently negative [19]. A massive, heterogeneous renal cortical mass with macroscopically visible fat is a reliable sign of AML and is therefore considered ‘classic AML’, but up to 5% of AML contains minimal fat, cannot be reliably diagnosed by imaging and is considered ‘fat-poor AML’ [20]. Atypical AML is composed predominantly of a single cellular component, with other elements present only in minimal amounts. The epithelioid variant (EAML) is characterized by numerous epithelioid muscle cells with abundant eosinophilic, granular cytoplasm and a paucity or absence of adipose tissue. Importantly, EAML must be differentiated from RCC, as it can closely resemble RCC both histologically and radiologically, leading to potential misdiagnosis [15]. Crucially, fat-poor AMLs tend to have a slower growth rate (<5 mm/year) than RCC. In addition, AMLs typically do not compress surrounding structures, which is more characteristic of cancer [6].

**Figure 2:**
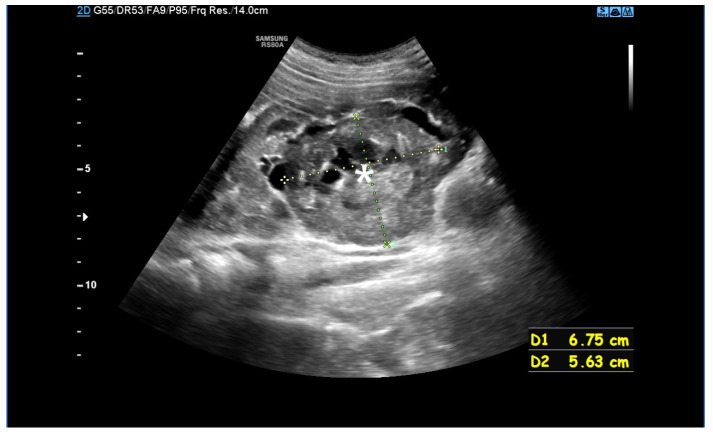
An ultrasound of a 10-year-old girl. A well-defined heterogeneous lesion, likely an AML, extending beyond the outline of the kidney. Within the lesion a fluid-filled area, likely a site of hemorrhage (*).

Potential complications of AML include haemorrhage (Wunderlich syndrome), the risk of which increases with the size and vascularisation of the lesion (Figure 2), and mass effect, which can cause discomfort or pain and may impair renal function by compressing urinary outflow or distorting normal renal parenchyma [19].

The main criteria for surgical intervention are symptomatic lesions, size greater than 4 cm and suspected malignancy. Malignant hemangiomas are extremely rare. Risk factors for aggressive behavior include absence of fat on imaging, clear epithelial histology, lesion size greater than 7 cm, and evidence of intravascular necrosis [4]. Identification of renal hemangiomas is based on diagnostic imaging. In most cases, biopsy is not necessary to make a diagnosis but may be required to exclude malignancy in difficult or complex cases, such as invisible fatty renal mass lesions.

### 3.2. Renal Cysts

Renal cysts occur in approximately 40% of children with TSC under the age of 5 years. The mTOR pathway is strongly associated with primary cilia-associated cystogenesis [12]. Studies of TSC1 or TSC2-associated renal cystogenesis in mice have shown that cystogenesis can be attributed to specific segments of the nephron, and that all tubular segments are involved in TSC cyst formation in mice [21]. Small renal cysts can be detected from infancy, and—similar to renal AML—their size and number tend to increase with age.

Cysts in TSC can arise throughout the nephron and may range from microscopic glomerular and cortical lesions to a polycystic phenotype or multicystic malformations resembling autosomal dominant polycystic kidney disease. Complications of renal cystic hyperplasia include infection, hemorrhage, pain, hypertension, and renal insufficiency. However, typical simple TSC cysts are usually asymptomatic and rarely problematic. The genetic and environmental mechanisms of cystogenesis remain poorly understood, and disease progression is highly variable. Classification of TSC-associated cystadenomas is still evolving. Bissler and Kingswood have proposed three cyst types associated with poor outcomes (microcystic, polycystic, and medullary polycystic) and two with less significant clinical impact (focal cystic and cortical cystic) [22].

### 3.3. Polycystic Kidney Disease in Children with Tuberous Sclerosis Complex

**Figure 3:**
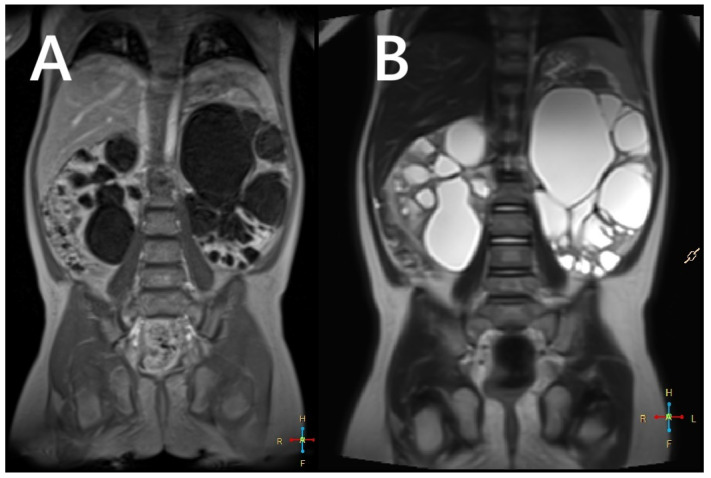
An MRI without (**A**) and with a contrasting agent (**B**) of a 3-year-old boy with a contiguous gene deletion syndrome involving TSC2/PKD1. Both kidneys are markedly enlarged, containing numerous thin-walled cysts of varying shapes. The remaining renal parenchyma is severely deformed.

In 2–3% of patients with TSC, TSC2/PKD1 contiguous gene syndrome (CGS) is found as a distinct disease entity with clinical features typical of TSC combined with severe and early polycystic kidney disease, almost always associated with early progression to end-stage renal disease (Figure 3). The TSC2 and PKD1 genes lie in a slightly overlapping tail-tail position on the short arm of chromosome 16 (16p13.3), and the cause of the disease is a large deletion involving the 3′ end of TSC2 and extending to the neighbouring PKD1, encoding polycystin-1. There appears to be a spectrum of variability in disease severity and prognosis for CGS, reflecting allelic variation and tissue mosaicism.

As with ADPKD, subtle cortical hyperechogenicity and renal enlargement may be evident on prenatal ultrasound in patients with CGS. Similarly, an infant or young child with TSC who presents with hypertension associated with enlarged, cystic kidneys may also have CGS. In milder cases, only genetic testing can distinguish the polycystic TSC phenotype from the CGS phenotype [23]. In addition to the potentially severe cystic phenotype, patients with CGS are also predisposed to developing renal masses due to TSC2 mutations. These masses present the same challenges as in TSC patients without PKD1 mutations. Consequently, renal imaging in CGS should follow the stricter TSC guidelines, recommending annual MRI or CT, rather than the protocols used for ADPKD [24].

### 3.4. Renal Cell Carcinoma

RCC (renal cell carcinoma) occurs in approximately 2–5% of patients with TSC [25]. However, the median age of diagnosis of RCC in TSC is approximately 28 years, while the average age in the general population is 53 years. The incidence of RCC in patients with TSC increases with age and affects children [26,27]. The course of RCC in patients with TSC appears relatively benign [28]. Interestingly, RCC in this group shows a female predominance of 2:1, in contrast to the male predominance observed in the general population [29]. RCCs associated with TSC tend to be multifocal and bilateral. For reasons yet to be elucidated, mutations of the TSC1 gene are overrepresented in this subgroup of patients compared to the general TSC population [30].

In addition to de novo growth of solid carcinomas, malignant transformation may occur in simple cysts. Predictors of malignant transformation of a cyst in RCC include calcification, wall thickening or septal enhancement. Regional enlarged lymph nodes and potential infiltration of the tumor into the renal blood vessels or perinephric space are also assessed. The Bosniak classification, based on sporadic lesions identified in moderate-risk adult cohorts without predisposing conditions, helps to objectively describe and assess the CT appearance of renal cysts and malignant potential and informs recommendations for further management [31,32]. This classification has not been formally validated in either pediatric or TSC patients and is speculated not to accurately reflect the potential risk of malignant transformation in these populations [32]. The use of the Bosniak classification is problematic due to the much higher baseline incidence of renal cysts and AML in patients with TSC. AML-type lesions without visible lipids may be classified as Bosniak lesions requiring surgery. In the TSC population with an increased risk of early renal failure, a percutaneous or open biopsy of suspicious lesions could be used as a nephron-sparing strategy.

### 3.5. Other Manifestations

Oncocytomas are benign lesions, histologically described as containing nests and tubular structures lined with cells with eosinophilic, granular cytoplasm. They are described as containing areas of a swollen mucinous or hyalinized stroma, with scattered tubular structures. Clear cytoplasm may be focally present in lesions with round nuclei and areas of degenerative cytological atypia. These features can produce clusters of tumor cells with enlarged nuclei, irregular nuclear contours, and smeared chromatin. In the general population, oncocytomas are usually present as unilateral, solitary lesions, whereas in patients with TSC they are often multiple and bilateral.

Predictive biomarkers remain poorly defined. The most used immunostains include cytokeratin 7, cyclin D1, kidney-specific cadherin, S100A1, and E-cadherin, with additional membrane positivity for CD117. Oncocytomas are negative for melanocytic markers. Notably, renal oncocytoma occurs more frequently in patients with TSC than in the general population. Immunohistochemistry is essential for diagnosis to avoid misclassification as epithelioid AML.

## 4. Blood Pressure in Children with Tuberous Sclerosis Complex

The primary cause of hypertension in patients with TSC is the formation of cysts or angiomyolipomas (AML) in the renal parenchyma [33]. The prevalence of hypertension among patients with TSC is higher than in the general population and increases with age, peaking in the fifth decade of life [34]. In the pediatric population, the prevalence of hypertension ranges from 5.7% (TOSCA study) to 23% (multicenter study from Belgium) [35,36].

Cysts and AML that appear and increase with age, as well as surgical interventions, lead to a reduction in the number of functional nephrons, which may result in increased renal blood flow to the remaining normal renal parenchyma, potentially leading to renal hyperfiltration and the development of hypertension. Renal hyperfiltration is defined as an elevated GFR in the range of 130 to 140 mL/min/1.73 m^2^ [37].

According to another hypothesis, haploinsufficiency of TSC1/TSC2 genes causing mTOR pathway overactivity may lead to glomerular hypertrophy and hyperfiltration; however, the mechanisms linking glomerular hyperfiltration to hypertension in TSC are not well understood [38]. Early diagnosis of hypertension in patients with TSC concerns cases of contiguous gene syndrome (CGS). In these patients, hypertension is detected in infancy, and renal function declines rapidly due to loss of active renal parenchyma [39]. The vascular malformations (MAS, renal artery stenosis) are rarely the cause of resistant hypertension. There is no evidence of an effect of antiepileptic treatment on blood pressure [40].

In the case of diagnosis and subsequent treatment of hypertension according to the ERKNet recommendations for TSC in children with TSC, the general guidelines for the management of hypertension in CKD should be followed. In children with elevated blood pressure (i.e., >95th percentile for age, sex, and height, and >120/70 mmHg in adolescents), a 24-h blood pressure measurement is recommended to be performed [41,42,43,44].

## 5. Nephrological Surveillance over Children with Tuberous Sclerosis Complex

Recommendations for nephrological surveillance and treatment of individuals with tuberous sclerosis complex (TSC) are included in the Updated International Diagnostic Criteria for Tuberous Sclerosis Complex 2021 and in the 2024 ERKNet Working Group recommendations [6,44].

Kidney imaging can be performed using three primary techniques: ultrasound, magnetic resonance imaging (MRI), and computed tomography (CT). While each modality has distinct mechanisms, advantages, and limitations, all play important roles in the diagnosis and surveillance of patients with TSC. However, their relative accuracy in this patient population has not yet been systematically compared [44,45,46]. The overview of imaging modalities in diagnosing and surveillance of TSC in children has been presented in Table 2.

Accurate assessment of blood pressure and kidney function (creatinine and cystatin C levels) is also important. At least once a year, clinical assessment of kidney function, proteinuria, and blood pressure is required in individuals with normal parameters. More frequent follow-ups should be performed with abnormal kidney function or hypertension. The frequency of abdominal imaging should be between one and three years, with annual scans required for tumors that are approaching 3 cm and/or appear to be growing. Longer follow-ups between imaging tests are acceptable when no tumors and only small tumors (<1 cm) with minimal growth over time are visible. A comparison of key aspects of the TSC is presented in Table 3 [6,44].

### 5.1. Renal Ultrasonography

**Figure 4:**
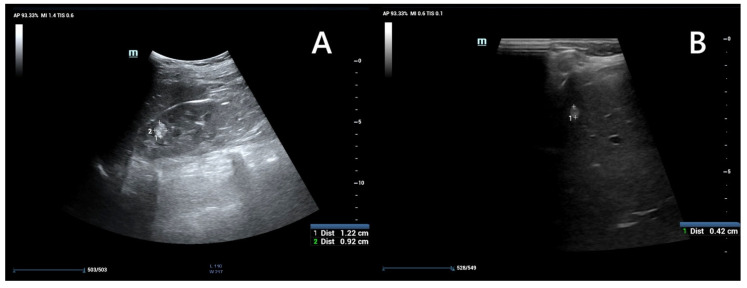
An ultrasound of a 13-year-old boy. Kidney with focal lesions with features suggestive of AMLs (**A**). Multiple oval hyperechoic lesions within the liver—most likely AMLs (**B**).

Ultrasound is widely recommended as a first diagnostic approach, helpful in detecting and monitoring lesions within kidney and other accessible parenchymal organs (Figure 4), and differentiating between their solid or cystic structure. When performed by an experienced operator, it is often sufficient for evaluating simple cysts; however in patients with large body mass, thick cystic septa, or not entirely anechoic lesion contents, further assessment with CT or MRI is recommended [44,47]. The kidney should be carefully scanned in both coronal and axial planes to ensure visualization of areas potentially obscured by artifacts, such as colonic gas or rib shadows [47].

AMLs in ultrasound typically appear as hyperechoic to renal parenchyma, homogeneous lesions, but their appearance can vary depending on the relative composition of fat, muscle, and vascular tissue [48]. The imaging appearance is non-specific, as up to 8% of renal cell carcinomas (RCCs) can also be hyperechoic, and confirmation by CT or MRI is recommended. However, it can provide valuable indicators: if the tumor is less than 3 cm and an acoustic shadow is present, angiomyolipoma is the most likely diagnosis. In contrast, the presence of a hypoechoic halo and intratumoral cysts suggests clear cell renal carcinoma (CCRC) [44,47,48]. Atypical or fat-poor AMLs require diagnostic evaluation with other, more advanced imaging modalities, as ultrasound images are varied and non-specific [48].

### 5.2. Magnetic Resonance Imaging

**Figure 5:**
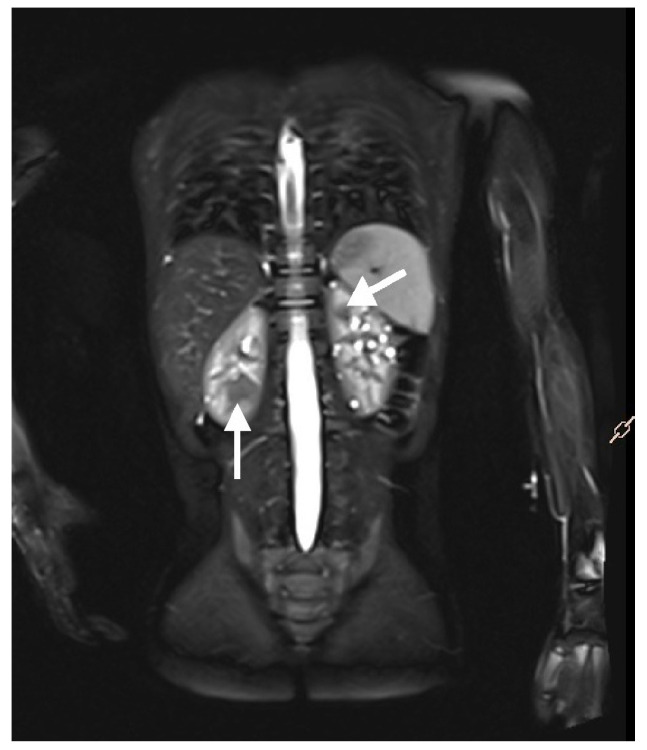
An MRI with contrast of a 5-year-old boy. Multiple solid focal lesions in both kidneys—atypical angiomyolipomas (arrows). Additionally, numerous small cysts within the parenchyma of both kidneys.

Magnetic resonance imaging (MRI) of the abdomen (Figure 5) and brain is the recommended imaging technique to diagnose and follow-up TSC-related kidney lesions. It remains the preferred method for evaluating AMLs, as 25–30% of them are fat-poor and are frequently overlooked in standard abdominal ultrasound examinations [6,49,50]. Additionally, MRI is highly effective in detecting other common abdominal abnormalities associated with TSC, such as renal cysts, aortic aneurysms, extrarenal hamartomas in the liver, and neuroendocrine tumors in the pancreas and other abdominal organs [51]. All individuals suspected of having TSC, should undergo MRI assessment of the brain to evaluate for cortical and subcortical tubers, subependymal nodules (SENs), neuronal migrational defects, and subependymal giant cell astrocytomas (SEGAs) [6].

The primary advantages of MRI are its lack of ionizing radiation, and its ability to provide excellent soft tissue contrast, even without the use of contrast agents. Compared to US, MRI provides more precise measurements of lesions and other pathological findings, particularly in cases involving coalesced or irregularly shaped lesions [47]. However, MRI has certain limitations, including reduced availability and the lower spatial resolution compared to CT, which avoids artifacts caused by abdominal movements that blur contours of lesions and organs [47]. Due to its stressful and time-intensive nature, MRI often requineedsral anesthesia, particularly in certain patient groups, including children, individuals with claustrophobia, or other anxiety or mental disorders. To reduce the necessity for multiple anesthesia sessions to facilitate diagnostic evaluation, MRI of the brain and abdomen is recommended to be performed in coordination [6,44].

In certain cases, contrast-enhanced MRI using gadolinium contrast agents proves to be a valuable tool, offering improved definition of angiomyolipomas and aiding in the differential diagnosis between fat-poor angiomyolipomas and other renal tumors. While the spatial resolution of contrast-enhanced MRI remains inferior to that of CT, it is often used for patients with allergies to iodinated contrast agents or those with a glomerular filtration rate (GFR) between 30 and 45 mL/min/1.73 m^2^ [47].

On MRI, a classic AML typically appears as a hyperintense fat component on both T1- and T2-weighted images, along with a hypointense component on T2-weighted images with fat saturation. A Black Boundary artifact, also known as an India ink artifact, appearing on the dual-sequence imaging is highly suggestive of AML [47,48,52].

MRI has proven useful for diagnosing atypical AML [47]; however, evidence indicates that imaging alone cannot definitively exclude RCC in cases of fat-poor solid kidney masses [53,54,55]. Definitive differentiation can only be achieved through histopathological examination. Fat saturation sequences in low-fat AML typically show no evidence of suppressed fat tissue.

The most common, hyperattenuating subtype presents similarly to smooth muscle. It is T1-hypointense, T2-hypointense, and usually enhances homogeneously in contrast-enhanced MRI modalities. Regions of signal loss on fat-suppressed sequences and chemical shift suppression are not observed. Similarly, isoattenuating AMLs are typically T2-hypointense; however, because of a higher fat cell content, they tend to show chemical shift suppression [48,53]. The AML with epithelial cysts subtype is characterized by smooth muscle tissue containing epithelial-lined cysts and minimal fat content. The epithelioid AML also exhibits minimal fat content, resulting in a wide range of imaging appearances that can make it indistinguishable from renal cell carcinoma (RCC) and other atypical AML. It is exceptionally rare, but it can be considered when a mass has small foci of macroscopic fat without calcification or when acute hemorrhage of a renal mass occurs [56].

### 5.3. Computed Tomography

Computed Tomography is usually the next preferred modality in case of the unavailability of MRI. Patients with normal renal function might profit from the use of intravenous contrast, which can help with the identification of renal cysts and fat-poor angiomyolipoma on abdominal CT [6,44].

The detection of classic AMLs relies on identifying regions containing fat cells within the kidney, evident as distinct areas with attenuation values below −10 Hounsfield units (HU). Detecting small amounts of fat may require performing CT scans with thin sections (<3 mm) and obtaining attenuation measurements using small regions of interest or even individual pixel values [48,53,57]. The fat-poor AMLs may be hyperattenuating (>45 HU, homogeneously enhancing), isoattenuating (between −10 HU and 45 HU) or contain epithelial cysts visible as cystic areas in the homogeneously enhancing, hyperattenuating media [48].

The appearance of AML can vary depending on the proportions of fat, vascular, and muscular tissue it contains. While RCC may also contain fat, it typically features calcifications, which can aid in differentiating it from AML. Similarly, on rare occasions, a liposarcoma may occur in the kidney and contain fat, potentially leading to a misdiagnosis as AML. To determine the renal origin of the tumour it is recommended to identify an accompanying defect in the renal parenchyma or the presence of a vessel extending into or through the renal parenchyma, as opposed to the renal hilum [53,58,59].

## 6. The Indications for Renal Biopsy in Children with Tuberous Sclerosis Complex

Tyburczy, M. E. Et Al. presented a genetic analysis of RCC in two patients with TSC and showed that these tumors developed independently of other renal lesions because of a so-called “second hit” in the TSC2 gene [60]. To rule out RCC or oncocytoma, in the case of rapidly growing lesions, especially fat-poor AML, a histopathological evaluation of the lesion should be considered. ERKNet recommendations for kidney involvement in TSC suggest biopsy in cases of fat-poor AML growing at a rate of at least 5 mm/year and/or unresponsive to mTOR inhibitor therapy, with surgical intervention indicated in cases of histopathologically confirmed RCC [44].

Histopathological examination of a kidney biopsy or nephron-sparing surgery commonly reveals spindle cells, epithelial cells, and vascular elements, typical for fat-poor AML. Staining of the biopsy sample with HMB-45 antigen (a monoclonal antibody specific for melanocyte cells) or cytokeratin allows AML to be distinguished from RCC and oncocytoma [5].

## 7. Treatment of Renal Manifestations of Tuberous Sclerosis in Children

### 7.1. Treatment of Arterial Hypertension and Role of Blockade of the Renin–Angiotensin–Aldosterone System

The risk of hypertension in patients with TSC is associated with the presence of renal lesions and possible surgical intervention. Infiltration of normal renal parenchyma by growing AML, hypertension, proteinuria, and hyperfiltration in patients with TSC also poses a risk for the progression of chronic kidney disease (CKD).

Renin–Angiotensin–Aldosterone system (RAAS) inhibitors (ACE inhibitors or angiotensin receptor blockers), recommended by ERKNet as first-line treatment for hypertension associated with TSC, are also effective in treating glomerular hyperfiltration and proteinuria [35,44].

In combination therapy with mTOR inhibitors and ACE inhibitors, a significant increase in the incidence of angioedema was observed in the group of kidney transplant recipients. These data suggest that consideration should also be given to initiating treatment with angiotensin receptor blockers (ARBs) in children with TSC treated with mTOR inhibitors [61].

### 7.2. The Role of mTOR Inhibitors in Treating Renal Lesions in Children with Tuberous Sclerosis Complex

In the pharmacological treatment of AML lesions, mTOR inhibitors (mammalian target of rapamycin) are recommended as first-line therapy. The mammalian target of rapamycin is a serine/threonine kinase belonging to the phosphoinositide-3-kinase (PI3K) family of related kinases (PIKK). The kinase occurs in two complexes, mTORC1 and mTORC2, and is involved in cell growth, proliferation, metabolism, and survival. The uncontrolled activity of this kinase is closely linked to the development of symptoms characteristic of TSC.

A meta-analysis and other observational studies have demonstrated a reduction in the number of surgical interventions (nephrectomy, embolization of lesions) and a lower risk of progression of chronic kidney disease during treatment with mTOR inhibitors [62,63]. Inhibition of the mTOR pathway affects not only the size and growth rate of TSC-associated AMLs, but also the intra-tumor aneurysms, which pose a high risk of bleeding [64]. To date, one retrospective study has been published describing improvement in cyst burden in terms of total number of cysts, sum of cyst diameters, and total cyst volume. Preclinical studies also show beneficial effects of mTOR inhibitors in ADPKD, but inconclusive results may suggest varying mTOR pathway activity in the development of renal cysts [65].

According to recommendations, mTOR inhibitors should be used for angiomyolipoma lesions larger than 3 cm in diameter. Prophylactic use of an mTOR inhibitor before a single lesion reaches a diameter of >3 cm may be beneficial in cases of rapid growth of angiomyolipomas (by >0.5 cm in diameter per year) and high overall mass of angiomyolipomas in the kidneys [44].

Among the substances available for treatment are rapamycin (sirolimus) and everolimus.

The EXIST randomized clinical trials evaluated the use of everolimus in the treatment of SEGA tumors and AMLs. The EXIST-1 trial evaluated the effect of everolimus on angiomyolipoma in pediatric patients with TSC treated for SEGA tumor [66]. In turn, the EXIST-2 trial observed the effect of everolimus on AML or LAM lesions. These studies demonstrated high efficacy in the treatment of AML lesions, which is consistent with earlier data on sirolimus, showing efficacy regardless of dose or minimum serum concentration. The established pediatric dosage of everolimus in children is 2.5 mg/m^2^/day [67,68].

No randomized trials have evaluated the use of sirolimus (known as rapamycin) in the treatment of renal TSC symptoms, but given its identical mechanism of action and data from non-randomized trials, sirolimus may be an alternative to everolimus, depending on availability. The established dosage of sirolimus in children is 0.5 mg/m^2^/day [44].

Drug dosages and side effects are summarized in Table 4 and Table 5.

Everolimus is well tolerated by children with TSC, and most adverse events occur within the first 6 months. Adverse events were classified as mild (grade 1 and 2) and treatment-interrupting (grade 3). The most common grade 1–2 adverse events associated with mTOR inhibitors in patients with TSC include aphthous stomatitis, irregular menstruation, hypercholesterolemia or hypertriglyceridemia, urinary tract infection, hypertension, acneiform dermatitis, insomnia, and interstitial lung disease. Proteinuria may also occur during mTOR therapy [69]. The most frequent grade 3 adverse events are irregular menstruation and aphthous stomatitis. A positive correlation between the occurrence of stomatitis and irregular menstruation with the dose of everolimus has also been observed [70].

The EXIST-1 study involving pediatric patients showed that the most common adverse reactions were stomatitis and oral ulcers. Furthermore, clinical data from this study do not indicate that everolimus affects growth and sexual maturation. No cases of nephrotoxicity have been reported in TSC patients exposed to everolimus, except for patients with severe renal impairment prior to treatment or patients who had undergone prior renal intervention [66]. Regular monitoring of electrolytes, glucose, liver, and kidney function during treatment with mTOR inhibitors is required in all patients [69].

Product information for mTOR inhibitors issued by the European Medicines Agency should include possible drug interactions. In the case of simultaneous use of an mTOR inhibitor and cyclosporine (condition after kidney transplantation in a patient with TSC), renal function should be closely monitored. Concomitant administration of mTOR inhibitors with strong CYP3A4 inhibitors (e.g., ketoconazole, voriconazole, itraconazole, telithromycin, clarithromycin) or CYP3A4 inducers (e.g., rifampicin, rifabutin) is not recommended. Concomitant administration of mTOR inhibitors and angiotensin-converting enzyme (ACE) inhibitors has also caused reactions such as angioedema. Notably, vaccinations may be less effective, and live vaccines are recommended to be avoided [71,72].

### 7.3. Renal Embolization

Image-guided transarterial selective arterial embolization (SAE) is most commonly utilized in patients presenting with tumor-related hemorrhage, allowing for detailed visualization of the tumor’s rich vascular supply and precise localization of active bleeding sites [73,74,75]. It serves as a valuable alternative to nephrectomy and is particularly advantageous in patients with a solitary functioning kidney, extensive bilateral renal involvement, or those who are not candidates for major surgery, as it enables the preservation of unaffected renal parenchyma [74]. Selective embolization has been shown to reduce tumor size as well as prevent further bleeding and loss of renal function in patients with renal AMLs [76]. Despite the often multifocal and progressive nature of AMLs in TSC patients, multiple endovascular embolization procedures can be performed safely and with acceptable risk profiles [77].

### 7.4. Surgical Treatment of Renal Lesions

**Figure 6:**
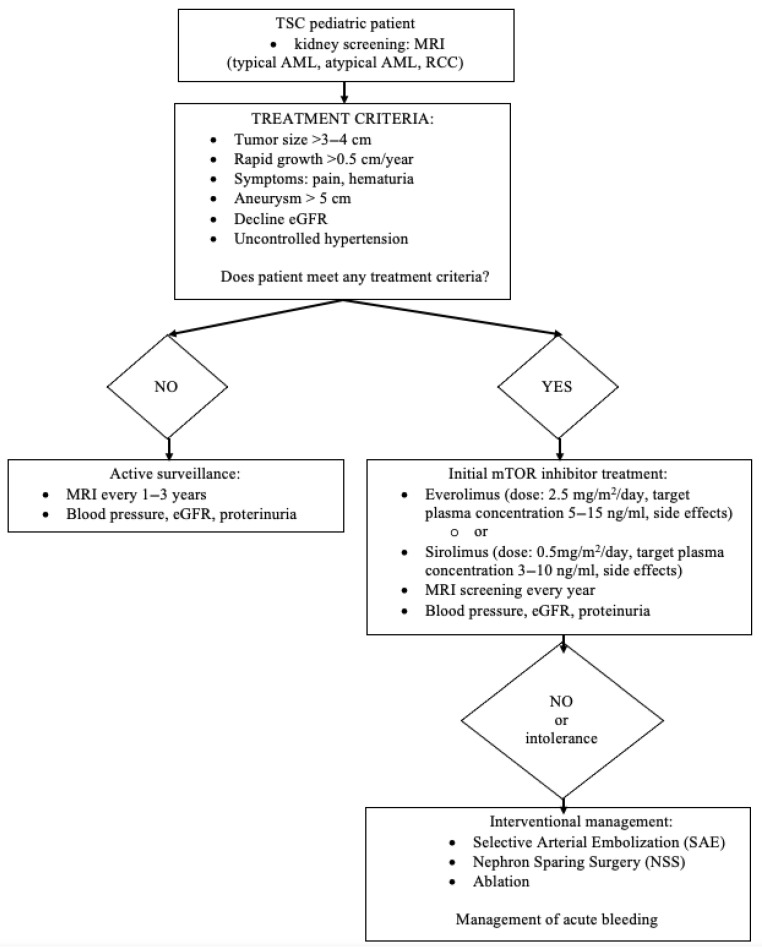
A decision tree: management and treatment of AML.

Nephron-sparing surgery (NSS) is the preferred treatment option when deciding on surgery for AML. In the case of RCC in patients with TSC, studies have shown that partial nephrectomy can prevent the development of CKD and the resulting morbidity and mortality. Patients with TSC may also present with AML and concomitant RCC, and tumor enucleation may still be appropriate. In cases of sporadic RCC, tumor enucleation, if technically feasible, yields oncological results comparable to those of tumor resection with a safety margin and is a parenchyma-sparing treatment option, even if malignancy is suspected. In practice, a combined procedure (enucleation resection) is usually performed. It should be noted that in cases of high probability of RCC, especially highly malignant RCC, resection with a safety margin should be considered [44]. Figure 6 shows a decision tree: management and treatment of AML.

## 8. Future Directions and Unanswered Questions in Patients with TSC

### 8.1. Renal Artery Stenosis and Mid-Aortic Syndrome in Children with Tuberous Sclerosis Complex

Hypertension in patients with TSC is more often associated with renal parenchymal disease, but vascular disease associated with obstruction or stenosis should also be considered. Renal artery stenosis (RAS) and middle aortic syndrome (MAS) may also occur as causes of hypertension in patients with TSC. RAS is a heterogeneous disease characterized by narrowing of the renal arteries. MAS, often referred to as abdominal coarctation, affects the abdominal aorta, renal arteries, and mesenteric arteries.

The pathogenesis of vascular disease in TSC is unknown. Dysplastic and degenerative changes, fibrocytic and myofibrocytic intimal proliferation, medial hyperplasia, and obliterative dysplasia within the arterial walls have been described. Patients with TSC and hypertension or symptoms suggestive of vascular stenosis should undergo whole-body screening or targeted vascular examination using duplex ultrasound, magnetic resonance angiography, and computed tomography angiography. Angioplasty is a treatment for stenotic lesions in TSC [45,46].

### 8.2. Other Therapeutic Options

Tolvaptan, a selective arginine vasopressin type 2 receptor antagonist, is the first approved treatment for autosomal dominant polycystic kidney disease (ADPKD) that targets the mechanism directly contributing to cyst development and growth. Despite evidence of beneficial treatment with tolvaptan in patients with ADPKD, there are no current data on the use of tolvaptan in patients with TSC with a significant cyst burden [78,79].

A so-called second generation of mTOR inhibitors (known as mTOR kinase inhibitors or TORKinibs) has been developed. The main difference from the first generation of mTOR inhibitors (sirolimus and everolimus) is that they have the ability to directly inhibit the kinase by blocking the ATP catalytic site, rather than binding FKBP12, resulting in inhibition of both the mTOR1 and mTOR2 pathways. If this dual inhibition allows for greater efficacy compared to the first generation of mTOR inhibitors without further significant toxic effects, these new agents may open new possibilities in the treatment of tuberous sclerosis [80].

## 9. Coordination of Care for Patients with TSC

Due to the multisystem manifestation of TSC, coordination of patient care should be sought. Although organ involvement changes with age, patients with TSC should receive multidisciplinary care from pediatric age onwards. The involvement of pediatricians, pediatric nephrologists, and nephrologists is of great importance in educating patients and their families and enabling appropriate intervention as the disease progresses. Every patient with TSC-related kidney changes has chronic kidney disease (CKD) of at least stage 1. It is recommended that the family of the TSC patient be included in the care plan and that a protocol for transition to adult care be planned [6,44]

## 10. Conclusions

Tuberous sclerosis complex (TSC) is a genetic disorder that affects many organ systems, especially the kidneys, where it can lead to complications such as angiomyolipomas, renal cysts, and renal cell carcinoma. The diagnosis requires clinical evaluation based on current diagnostic criteria, non-invasive imaging, and genetic testing.

Regular nephrological monitoring, including imaging studies and clinical evaluation in patients with TSC, is crucial for the early detection and treatment of complications such as bleeding or chronic kidney disease.

Treatment typically involves mTOR inhibitors, which effectively treat renal lesions and may reduce the need for surgery, although they can cause side effects that require monitoring. Ongoing research aims to better understand TSC and explore new treatment options. A multidisciplinary approach is critical to optimizing care and outcomes for children affected by this disease.

## Figures and Tables

**Table 1:** Updated diagnostic criteria according to the 2021 International Tuberous Sclerosis Complex Consensus Group [6].

**Updated international TSC diagnostic criteria** Definite TSC: 2 major features or 1 major feature with 2 minor features Possible TSC: 1 major feature or at least 2 minor features Genetic diagnosis: A pathogenic variant in TSC1 or TSC2 is diagnostic for TSC
**Major criteria** Skin: Hypomelanotic macules (at least 3; at least 5 mm diameter) Shagreen patch Head: Angiofibroma (at least 3) or fibrous cephalic plaque Fingers and toes: Ungual fibromas (at least 2) Eyes: Multiple retinal hamartomas Brain: Multiple cortical tubers and/or radial migration lines Subependymal nodule (at least 2) Subependymal giant cell astrocytoma Heart: Cardiac rhabdomyoma Lungs: Lymphangioleiomyomatosis (LAM) * Kidneys: Angiomyolipomas (at least 2) *
**Minor criteria** Skin: “Confetti” skin lesions Teeth: Dental enamel pits (at least 3) Gingiva: Intraoral fibromas (at least 2) Eyes: Retinal achromic patch Kidneys: Multiple renal cysts Liver, spleen, and other organs: Nonrenal hamartomas Bones: Sclerotic bone lesions

* A combination of the 2 major clinical features LAM and angiomyolipomas without other features does not meet the criteria for a definite diagnosis.

**Table 2:** The overview of imaging modalities in diagnosing and surveillance of TSC in children [6,47].

Technique	Advantages	Disadvantages
Ultrasound	Convenience Accessibility Lack of ionizing radiation	Low precision Poor reproducibility of results
CT	Excellent spatial resolution	Ionizing radiation Reactions to iodinated contrast media
MRI	Excellent soft tissue contrast Lack of ionizing radiation	Often requires general anesthesia Spatial resolution inferior to CT

**Table 3:** Comparison of key aspects in the TSC [6,44].

	TSC Guidelines 2021 (Northrup, Hope Et Al.) [6]	ERKNet 2024 [44]
Genetic testing	recommended for genetic counseling purposes or when the diagnosis of TSC is suspected or in question but cannot be clinically confirmed (category 1)	recommended for all patients with TSC (level X, strong) mid
Hypertension	annual clinical assessment blood pressure (category 1) inhibitor of the renin-aldosterone-angiotensin system as first-line therapy (category 1)	annual standardized office blood pressure assessment (level B, strong), twenty-four-hour ambulatory blood pressure monitoring in children (≥5 years) (level B, moderate) use of angiotensin-converting enzyme inhibitors or angiotensin receptor blockers as the first-line treatment (level B, strong)
Monitoring of kidney function	annual clinical assessment of kidney function (creatinine-based eGFR, cystatin C-based eGFR) and proteinuria (category 1)	annual assessment of AML -related complications (pain, risk bleeding) and biochemical tests (creatinine-based eGFR, cystatin C-based eGFR) (level B, moderate)
Kidney imaging	kidney imaging at the time of TSC diagnosis (category 2A) frequency of imaging should be 1–3 years (category 2A) MRI is the preferred imaging modality (category 2A)	kidney imaging at the time of TSC diagnosis (level B, strong) imaging follow-up of the kidneys at intervals of 1–3 years (level B, strong). MRI is the preferred imaging technique to diagnose and follow-up TSC-related kidney tumours (level B, strong)
mTOR treatment	mTOR inhibition in AML of >3 cm (category 1) everolimus dose ≤ 5 mg daily (category 1)	mTOR inhibition in AML of >3 cm (level A, strong) preventatively using mTOR inhibitor in cases of rapid AML growth (>0.5 cm per year in diameter) or high overall AML burden in the kidneys (level D, weak) everolimus starting dose 2.5 mg/m^2^/day (level D, weak) sirolimus is a reasonable alternative to everolimus
Surgical intervention	for acute AML, hemorrhage embolization is more appropriate (category 2A)	actively bleeding AML of any tumour size is an accepted indication for arterial embolization (level X, strong)
Management of patients with TSC	multispecialty medical teams for the care	multidisciplinary follow-up (level C, moderate)

**Table 4:** Dose and target plasma concentration of mTOR inhibitors.

	Dose [mg/m^2^/day]	Target Plasma Concentration [ng/mL]
Everolimus	2.5	5–15
Sirolimus	0.5	3–10

**Table 5:** Adverse events caused by mTOR inhibitors.

Adverse Events Caused by mTOR Inhibitors in Patients with TSC	
Grade 1, 2 (Mild)	Grade 3 (Treatment-Interrupting)
hypercholesterolaemia	aphthous stomatitis
hypertriglyceridaemia	irregular menstruation
urinary tract infection	
hypertension	
dermatitis acneiform	
insomnia	
interstitial lung disease	

## Data Availability

No new data were created or analyzed in this study. Data sharing is not applicable to this article.

## Abbreviations

The following abbreviations are used in this manuscript:

ACE	Angiotensin-Converting Enzyme
ACMG	American College of Medical Genetics
ADPKD	Autosomal Dominant Polycystic Kidney Disease
AML	Angiomiolipoma
ATP	Adenosine Triphosphate
ARB	Angiotensin Receptor Blocker
CCRC	Clear Cell Renal Carcinoma
CD117	Cluster of Differentiation 117
CGS	Contiguous Gene Syndrome
CKD	Chronic Kidney Disease
CYP3A4	Cytochrome P450 3A4
EAML	Epithelioid Angiomyolipoma
EMA	European Medicines Agency
ERA	European Renal Association
ERKNet	European Rare Kidney Disease Reference Network
FKBP12	FK506 Binding Protein 12
GFR	Glomerular Filtration Rate
HMB-45	Human Melanoma Black-45
HU	Hounsfield Unit
LAM	Limfangioleiomiomatosis
MAS	Mid-Aortic Syndrome
MRI	Magnetic Resonance Imaging
NSS	Nephron-sparing surgery
mTOR	mammalian Target Of Rapamycin
PanNET	Pancreatic Neuroendocrine Tumor
PEC	Perivascular Epithelial Cell
PECOMA	Perivascular Epithelioid Cell Tumor
PI3K	Phosphoinositide 3-Kinase
PIKK	PI3K-Related Kinases
PKD	Polycystic Kidney Disease
RAAS	Renin–Angiotensin–Aldosterone System
RAS	Renal Artery Stenosis
RCC	Renal Cell Carcinoma
RTA	Renal Tubular Acidosis
SAE	Selective arterial embolization
SEGA	Subependymal Giant Cell Astrocytoma
SEN	Subependymal Nodule
TAND	TSC-Associated Neuropsychiatric Disorder
TORKinibs	mTOR Kinase Inhibitors
TSC	Tuberous Sclerosis Complex
US	Ultrasound
